# An ethnopharmacological assessment of medicinal plants in Malanje Municipality, Angola

**DOI:** 10.3389/fphar.2025.1702353

**Published:** 2026-02-25

**Authors:** Agostinho António Barroso, André Alberto Martins, Agostinho Morais, Peterson Carlos Foguete Katenda, Madalena Feca Jamba, Mateus Ferreira Alfredo Gonçalves, Mateus André Sebastião, Bernardo Nicodemo Chimbuco, Yanelis Saucedo Hernández, Dany Siverio Mota, Venancio Ribalta Ribalta, Amandio Gomes, Enoel Hernándes Barreto, Eduardo Ekundi-Valentim

**Affiliations:** 1 Instituto Politécnico, Universidade Rainha Njinga A Mbande, Malanje, Angola; 2 Universidade Rainha Njinga a Mbande, Malanje, Angola; 3 Departamento de Farmacia, Facultad de Química y Farmacia, Universidad Central “Marta Abreu” de Las Villas, Santa Clara, Villa Clara, Cuba; 4 Departamento de Biologia, Faculdade de Ciências Naturais da Universidade Agostinho Neto, Luanda, Angola

**Keywords:** indigenous knowledge, traditional healers, biodiversity conservation, rank order priority, Terminalia

## Abstract

**Background:**

Malanje Municipality in north-central Angola harbors exceptional botanical and cultural diversity, yet remains poorly documented for traditional medicinal plant knowledge; this study provides the first systematic ethnopharmacological baseline to guide pharmacological prioritization, conservation, and policy-relevant integration of traditional medicine.

**Methods:**

Between 2018 and 2023, we conducted semi-structured interviews (n = 20 traditional healers), participatory observation, *in situ* photographic documentation, and GPS mapping. Voucher specimens were taxonomically verified against herbarium material and online resources. Quantitative indices included frequency measures and rank order priority (ROP); therapeutic indications were grouped using ICD-11 categories.

**Results:**

Informants reported 272 ethnospecies, of which 78 taxa (39 families) were identified to species level. Fabaceae (9%), Asteraceae (6.4%), and Euphorbiaceae, Poaceae, and Zingiberaceae (each 5.1%) were most represented families. Leaves (53.8%) and roots (42.3%) were the principal parts used; decoction (60%) and maceration (31%) were the most common preparations. ROP prioritized *Terminalia brachystemma* (81.8), *Securidaca longepedunculata* (54.4), and *Mondia whitei* (52.2) for follow-up study. Treated conditions clustered in gastrointestinal disorders (43.6%) and infectious/parasitic diseases (29.5%). Healers reported several contraindications and observable adverse effects.

**Conclusion:**

This work provides the first comprehensive ethnopharmacological register for Malanje Municipality, highlighting high-priority species for phytochemical, pharmacological, and toxicological evaluation and identifying conservation and sustainable-use concerns (notably root harvest). Limitations include a modest sample of informants and incomplete taxonomic resolution for many ethnospecies.

**Recommendations:**

Expand sampling across the province, complete voucher identification, perform contamination and toxicity screening, and develop community-led cultivation and stewardship plans that align with Angola’s National Policy for Traditional and Complementary Medicine.

## Introduction

Medicinal plants have been central to human progress, forming the backbone of traditional therapies and contributing significantly to modern pharmaceuticals (Dar et al., 2017). Angola, blessed with rich botanical diversity, boasts approximately 6850 native plants, including 997 endemic species (Goyder et al., 2019). Despite their profound economic, commercial, and health benefits, ethnobotanical research remains sparse. Encouragingly, recent studies are beginning to uncover how the Angolan populace traditionally uses these medicinal plants (Göhre et al., 2016; Heinze et al., 2017; Heinze et al., 2019; Lautenschläger et al., 2018; Urso et al., 2016). These investigations, conducted mainly in the northern and southern regions, underscore both the richness of Angola’s ethnobotanical heritage and the urgent need to expand research to underrepresented areas, such as Malanje.

Herbs and medicinal plants occupy a vital place in the cultural and traditional practices of African societies. In Angola, this legacy is reflected in a vast repertoire of culinary, ritual, and healing traditions developed to manage a wide range of ailments. Knowledge of these natural resources is predominantly transmitted orally through storytelling, apprenticeship, and communal experience (Heinze et al., 2017; Malik et al., 2018; Tchamba, 2019). However, the oral nature of this knowledge has contributed to a scarcity of written records, limiting the documentation and scientific validation of species diversity, their pharmacological roles, and their cultural importance. Preserving and studying these natural resources is therefore essential not only for safeguarding indigenous knowledge systems but also for promoting their transformation into standardized natural products that can strengthen healthcare systems and stimulate bioeconomic development (Mendonça et al., 2015).

Across Africa, many nations have adopted World Health Organization (WHO) guidelines encouraging the integration of traditional medicine into formal health systems (WHO, 2013). Angola took an important step in this direction on October 2, 2020, by approving the National Policy for Traditional and Complementary Medicine through Presidential Decree No. 253/20. Angola (2025): this landmark policy promotes the coexistence of evidence-based traditional practices and modern medicine, encouraging scientific research to evaluate their safety, efficacy, and socio-economic impact. Ethnopharmacological research is thus recognized as a key instrument for effectively implementing this policy (Angola, 2025).

Historical accounts, such as those documented by Tchamba et al. (2024), highlight the pivotal role of 19th-century Christian missionaries in cataloging the medicinal flora of Angola, thereby laying the early foundations for ethnopharmacological inquiry in the region. These early records attest to the longstanding integration of plant-based remedies into local healing systems, a tradition that remains vibrant and culturally embedded.

In recent years, scientific investigations have further illuminated the pharmacological potential of key Angolan species. For example, Samba et al. (2025) conducted an in-depth phytochemical and pharmacological evaluation of *Cochlospermum angolense*, demonstrating notable antioxidant activity and reinforcing the therapeutic prospects suggested by traditional use. Such findings underscore the importance of bridging ancestral knowledge with contemporary analytical approaches to validate, strengthen, and sustainably harness indigenous plant resources.

Studies from surrounding provinces provide additional methodological and contextual grounding. In Uíge, surveys by Lautenschläger et al. (2018) and Göhre et al. (2016) documented a rich repertoire of medicinal species used by Bakongo communities, particularly in disturbed savannah ecosystems where ecological pressure intersects with cultural resilience. Similarly, Heinze et al. (2017) and Heinze et al. (2019) examined the economic and medicinal relevance of native flora in Cuanza Norte, emphasizing the dual role these plants play in both primary healthcare and household livelihoods.

Further south, Urso et al. (2016) investigated Mopane woodland communities, revealing extensive knowledge of wild plants used for medicinal and nutritional purposes. In Bié Province, Novotna et al. (2020) described the practices of root doctors, traditional healers whose treatments draw on deep ethnomedical expertise and an intimate understanding of local biodiversity. Collectively, these regional studies attest to the remarkable diversity and depth of Angola’s ethnobotanical landscape, highlighting its scientific relevance, cultural significance, and the need for continued, systematic documentation.

Despite these advances, Malanje remains conspicuously understudied, leaving a significant gap in understanding regional patterns of medicinal plant use. The province occupies a transitional ecological zone between Guineo-Congolian forest influences and central-eastern savannah ecosystems, suggesting high potential for botanical and cultural diversity. The main objective of this study is to contribute to filling that gap by documenting and analyzing the ethnopharmacological knowledge of traditional healers in Malanje Municipality.

Beyond increasing Angola’s ethnopharmacological baseline, it aims to identify species with potential pharmacological and socio-economic importance, help the preservation of indigenous knowledge, and support sustainable biodiversity management; ultimately, this research provides a vital foundation for future surveys, pharmacological validation, bioprospecting efforts, and policy initiatives aimed at integrating traditional medicine into national healthcare and biodiversity conservation strategies.

## Materials and methods

The research was carried out through several stages: conducting a bibliographic study to select the appropriate methodology, selecting and contacting informants, performing interviews, engaging in participatory observation to identify the reported ethnospecies, and organizing, processing, and analyzing the collected data.

### Local of study

**FIGURE 1:**
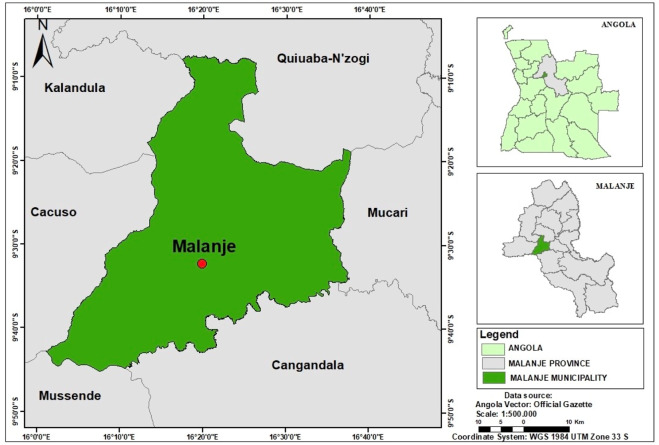
Study area map. Location of Malanje province in Angola and the study area in the municipality of Malanje, kindly provided by Pereira (2023).

The study was conducted in the Municipality of Malanje, located in Malanje Province, north-central Angola. The municipality covers an area of approximately 2,422 km^2^ and lies at an average elevation of 1,122 m above sea level, between coordinates 9°32′24.54″S and 16°20′27.46″E, as show the Figure 1 (Huntley, 2023). It has an estimated population of approximately 221,275 inhabitants. In the Köppen classification, the study area has a tropical rainforest climate, characterized by a well-defined rainy season lasting 6–7 months (from October to April), with precipitation between 900 and 1,200 mm, and a dry season of 5–6 months (from May to September). Average annual temperatures range from 20 °C to 25 °C, and relative humidity varies between 65% and 75%. Soil types in Malanje vary regionally: the northern areas are dominated by Ferralsols and Acrisols, while the central and southern zones are characterized by Luvisols, with transitional areas showing intergrades with Cambisols (Huntley, 2023; Pereira, 2023).

The municipality borders Kiwaba Nzogi to the north, Mucari to the east, the municipalities of Cangandala and Mussende (Cuanza Sul Province) to the south, and Cacuso and Calandula to the west. Malanje Province is predominantly inhabited by members of the Kimbundu ethnolinguistic group, whose language, Kimbundu, is widely spoken across both rural and urban contexts. Despite this demographic predominance, the province exhibits considerable ethnolinguistic heterogeneity, with minority populations such as the Bakongo, Chokwe, and Umbundu also present.

Population: A total of twenty (20) traditional healers participated in the survey, selected through convenience sampling. With the assistance of the local Association of Traditional Healers of Malanje, participants were identified and contacted. The healers practiced in diverse settings, with some based in local markets and others operating from dedicated herbalist shops, reflecting the typical contexts of traditional healing in the region.

### Ethical procedures

The Ethics Committee of the University Rainha Njinga a Mbande approved the project (0017/2018). Additional authorization from the local authorities was also obtained. The Code of Ethics of the International Society of Ethnobiology (International Society of Ethnobiology, 2006) was followed during the fieldwork.

### Registration of ethnobotanical information

The data were gathered through interviews and participatory observation (as evidenced in the attached survey form) to collect detailed information on the common and scientific names of plants, the parts used, processing methods, and the primary ailments treated. Additional data were collected on traditional healers practicing natural medicine with medicinal plants. With the assistance of healers familiar with the habitats of each species, *in vivo* photographs were taken, and GPS coordinates were recorded. A botanical specialist, Dr. Amândio Gomes, a coauthor, subsequently performed taxonomic identification by comparison with authenticated herbarium material, using dichotomous keys and specialized references (e.g., Conspectus Florae Angolensis and Flora Zambesiaca), and verified updated scientific names through online databases such as https://www.worldfloraonline.org and https://powo.science.kew.org) and by direct observation. Throughout the study, special emphasis was placed on conserving medicinal plant resources and preserving indigenous ethnobotanical knowledge. Community participation was encouraged to ensure that traditional healers and residents remain active custodians of both biological and cultural heritage, promoting sustainable use and intergenerational transmission of local knowledge. The conservation status of the collected plants was checked using the IUCN Red List of Threatened Species (IUCN, 2018).

### Data analysis

Plant species are categorized by their family, scientific name, common name, parts utilized, preparation methods, and therapeutic applications in order of significance. The ethnopharmacological data collected were quantitatively evaluated using the rank order priority (ROP) metric to gauge their healing potential.

The healing potential of a plant can be established by ROP and was calculated using the following formula (Siddique et al., 2021):

ROP=FL×RP. (1)

Here, FL is the level of fidelity, and RP is the relative popularity of the species in question.

The fidelity level (FL) is, in turn, determined using the following expression (Siddique et al., 2021).

$$\text{FL}=\left(\frac{Ip}{Iu}\right)\times 100.$$ (2)

Here, *Ip* is the number of informants who mentioned the species for the same principal purpose, and *Iu* is the total number of informants who mentioned the plant for any use.

The formula used for calculating relative popularity (RP) is

$$\text{RP}=\left(\frac{Iu}{Imc}\right),$$ (3)

where *Iu* is the number of informants who suggested a particular plant species and *Imc* is the number of informants who cited the most frequently cited plant species. RP takes values between 0 and 1. The most popular species have an RP value closer to 1, while the unpopular, or less known, have an RP value closer to 0.

According to Formulas 1, 2, the ROP can also be expressed as shown in Equations 4, 5 presented below:

$$\text{ROP}=\left(\frac{Ip}{Iu}\right)\times \left(\frac{Iu}{Imc}\right)\times 100.$$ (4)

Simplifying terms, it could be expressed as follows:

$$\text{ROP}=\left(\frac{Ip}{Imc}\right)\times 100.$$ (5)

Although the FL allows for estimating the healing potential of a species concerning its most cited use, it also makes it difficult to compare the relative importance with other medicinal plants with similar FL. For this reason, the ROP is a more appropriate parameter for assigning an order of curative potential in an ethnobotanical study. According to Equation 5, the ROP does not depend on the number of informants who cite the plant for any use, but on the number of times it is cited for the most frequent use and the number of times the most cited species is mentioned.

Botanical species with high ROP values have great curative potential. They are candidates for further studies, such as pharmacognosic, phytochemical, and biological assays, to evaluate their biological activity, therapeutic effectiveness, and toxicity.

The International Classification of Diseases (ICD-11) was used to group plants into different categories for medicinal purposes (WHO, 2019).

## Results and discussion

### Sociodemographic characteristics of the informants

The study involved 20 participants, 10 men and 10 women, who worked predominantly in the Cangambo market (13), with a smaller number working in traditional medicine stalls (5) and in the Ritondo neighborhoods (2). The predominance of healers operating in the Cangambo market, followed by those in the traditional medicine stalls and the Ritondo neighborhoods, highlights the central role of marketplaces as hubs for the circulation of knowledge, medicinal products, and therapeutic practices. Recent ethnobotanical research underscores that these spaces serve as strategic environments for the preservation and transmission of traditional knowledge, functioning as dynamic interfaces where healers and community members interact and exchange experiences (Lautenschläger et al., 2018; Heinze et al., 2017).

These healers were on average 51 years old, ranging from a young 26-year-old to a seasoned 75-year-old, with an impressive average of 25 years of experience in the field. Studies carried out in Uíge (Göhre et al., 2016; Lautenschläger et al., 2018) and southern Angola (Urso et al., 2016) indicate that knowledge of medicinal plants is cumulative and strongly shaped by lived experience. Educational attainment varied significantly: 55% were illiterate, 40% had completed primary education, and only 5% had secondary education. These educational levels are consistent with previous research in Angola (Urso et al., 2016) and highlight a trend within the community of traditional healers.

Gender representation among practitioners was balanced, with equal numbers of men and women involved. Women demonstrated greater familiarity with plants located near homes and villages, particularly those used for treating mild illnesses, preparing food, and caring for children (Torres‐Avilez et al., 2026). Men, in contrast, were more engaged with species cultivated on farms or collected in remote wilderness areas (Amorozo and Gély, 1988). As Lautenschläger et al. (2018) and Heinze et al. (2017) emphasize, gender functions as an organizing principle rather than a limiting factor: both men and women hold significant mastery, and their distinct specializations together reinforce the resilience and breadth of traditional medicinal knowledge.

The present study did not aim to examine how informants’ characteristics influence traditional knowledge. However, a survey carried out in communities in southern Angola did not show notable differences in this aspect (Urso et al., 2016).

Age and duration of experience working with medicinal plants lend greater expertise to traditional healers in using natural remedies to address health issues. In our study, 95% of the respondents were over 30 years old, suggesting a gradual replacement of individuals knowledgeable in traditional medicine. This highlights the urgent need for scientific research to document and preserve the wealth of information on medicinal plants in Malanje.

The high occurrence of illiteracy among participants indicates that oral transmission of knowledge plays a crucial role in traditional medicine in Malanje. This underscores the importance of documenting and preserving the wisdom held by these healers.

### Botanical identification, floristic richness, and relative popularity level

From the 20 interviews conducted in Malanje, an array of 272 ethnospecies of medicinal plants were cataloged (see Supplementary Material), all renowned for their healing properties. These plants are known by local names in Kimbundu, making botanical identification and cross-referencing with external sources challenging. Ethnobotanical wisdom varied widely among individuals; many plants were mentioned by only a handful of informants, highlighting a potential threat to preserving this traditional knowledge. A study in Bié province, Angola (Novotna et al., 2020), uncovered that 34 ethnospecies used in traditional medicine were mentioned by only a single informant. Comparable findings emerged from ethnobotanical research in Bibala Municipality, Namibe Province (Urso et al., 2016), where most plants were noted by just one or two individuals. Similarly, in the Cuanza Sul Province (Fançony, 2021), out of the 94 ethnospecies documented, 33 (34.74%) were noted by only one informant, while 26 (27.37%) were cited by two others. These instances might arise because herbalists and traditional healers view their wisdom as a sacred or economic secret, which they choose not to share with others in their field. A similar pattern emerged in a study conducted in Zimbabwe (Ngarivhume et al., 2015). These parallel findings suggest a common approach to the distribution of knowledge among practitioners, healers, and therapists of traditional medicine across various regions of Angola. This underscores the necessity of pursuing further ethnopharmacological research to document and preserve the rich knowledge held by different communities.

The multitude of documented plant species reveals that traditional medicinal practices leveraging natural products remain a prevalent choice for residents of this area. Through participatory observation, macroscopic analysis, and sample comparison with existing literature, 78 ethnospecies (28.7%) were successfully identified (Table 1). This highlights local expertise in therapeutic uses and underscores the necessity for botanical research to classify these plants taxonomically and thoroughly document the region’s botanical wealth.

**TABLE 1:** Plant identification and ethnopharmacological assessment (ROP).

No.	Family	Scientific name	Habitus	Common name	Distribution data for each species	Collecting place	ROP
1.	Combretaceae	*Terminalia brachystemma* Welw. ex Hiern	Tree/shrub	Mueia (Kimb.)	Native	Shrubland savanna	81.8
2.	Polygalaceae	*Securidaca longepedunculata* Fresen.	Shrub	Mutungo (Kimb.)	Native	Shrubland savanna	54.4
3.	Apocynaceae	*Mondia whitei* (Hook.f.) Skeels	Climbing	Mundondo (Kimb.)	Native	Cropland/forest gallery	52.2
4.	Cochlospermaceae	*Cochlospermum angolense* Welw. ex Oliv.	Shrub	Mbrututo (Kimb.)	Native	Shrubland savanna	34
5.	Rubiaceae	*Mussaenda arcuata* Poir.	Shrub	Kabolebole (Kimb.)	Native	Shrubland savanna	31.7
6.	Fabaceae	*Senna occidentalis* (L.) Link	Shrub	Mudia-nhoca (Kimb.)	Native	Disturbed places	29.4
7.	Zingiberaceae	*Aframomum alboviolaceum* (Ridl.) K.Schum.	Annual herb	Ginguenga (Kimb.)	Native	Shrubland savanna	27.2
8.	Annonaceae	*Monodora myristica* (Gaertn.) Dunal	Tree	Jipepe (Kimb.)	Native	Forest gallery	27.1
9.	Anacardiaceae	*Lannea welwitschii* (Hiern) Engl.	Tree	Mucumbi (Kimb.)	Native	Forest gallery	22.7
10.	Annonaceae	*Xylopia aethiopica* (Dunal) A.Rich.	Tree	Missae (Kimb.)	Native	Forest gallery	18.7
11.	Fabaceae	*Vachellia sieberiana* (DC.) Kyal. & Boatwr.	Tree	Musonge (Kimb.)	Native	Wooded savanna	18.1
12.	Burseraceae	*Canarium schweinfurthii* Engl.	Tree	Mubafo (Kimb.)	Native	Forest gallery	18.1
13.	Moringaceae	*Moringa oleifera* Lam.	Shrub	Moringa (port.)	Naturalized	Fallows/cropland	15.8
14.	Euphorbiaceae	*Ricinus communis* L.	Shrub	Jimono (Kimb.)	Naturalized	Disturbed places	15.8
15.	Rubiaceae	*Crossopteryx febrifuga* Benth.	Shrub	Mussesse (Kimb.)	Native	Shrubland savanna	15.4
16.	Poaceae	*Cymbopogon densiflorus* Stapf	Grass	Saco-saco (Kimb.)	Naturalized	Cropland	13.5
17.	Myrtaceae	*Eucalyptus globulus* Labill.	Tree	Eucalipto (Port.)	Cultivated	Fallows	11.3
18.	Fabaceae	*Bauhinia forficata* Link	Shrub	Pata de vaca (Port.)	Introduced	Disturbed places	11.3
19.	Amaranthaceae	*Dysphania ambrosioides* (L.) Mosyakin & Clemants	Annual herb	Santa Maria (Port.)	Naturalized	Disturbed places	11.3
20.	Fabaceae	*Eriosema psoraleoides* (Lam.) G.Don	Shrub	Kidiambuiz (Kimb.)	Native	Disturbed places	11.3
21.	Celastraceae	*Gymnosporia senegalensis* Loes.	Shrub	Mussambela (Kimb.)	Native	Shrubland savanna	11
22.	Asparagaceae	*Asparagus* sp.	Annual herb	Kanhanga (Kimb.)	Native	Shrubland savanna	11
23.	Amaranthaceae	*Beta vulgaris* L.	Annual herb	Beterraba (Port.)	Introduced	Cropland	6.8
24.	Euphorbiaceae	*Euphorbia pulcherrima* Willd. ex Klotzsch	Shrub	Bico de Papagaio (Port.)	Native	Arid savanna	6.8
25.	Zingiberaceae	*Aframomum melegueta* K. Schum.	Annual herb	Ndungo à congo (Kimb.)	Native	Forest gallery	6.7
26.	Zingiberaceae	*Zingiber officinale* Rosc.	Annual herb	Gingibre (Port.)	Cultivated	Cropland	6.7
27.	Amaryllidaceae	*Allium cepa* L.	Annual herb	Cebola (Port.)	Cultivated	Cropland	6.7
28.	Solanaceae	*Nicotiana tabacum* L.	Annual herb	Tabaqueiro (Port.)	Cultivated	Cropland	4.5
29.	Solanaceae	*Solanum lycopersicum* L.	Annual herb	Tomateiro (Port.)	Cultivated	Cropland	4.5
30.	Rutaceae	*Citrus limon* (L.) Burm. F.	Shrub	Limoeiro (Port.)	Cultivated	Cropland	4.5
31.	Phyllanthaceae	*Phyllanthus niruri* L.	Shrub	Quebra-pedra (Port.)	Native	Shrubland savanna	4.5
32.	Myrtaceae	*Psidium guajava* L.	Shrub	Goiabeira (Port.)	Cultivated	Cropland	4.5
33.	Meliaceae	*Azadirachta indica* A.Juss.	Tree	Neem (Port.)	Introduced	Disturbed places	4.5
34.	Lauraceae	*Persea americana* Mill.	Tree	Abacateiro (Port.)	Cultivated	Cropland	4.5
35.	Phyllanthaceae	*Hymenocardia acida* Tul.	Shrub	Mupexi (Kimb.)	Native	Shrubland savanna/miombo	4.5
36.	Costaceae	*Costus spiralis* (Jacq.) Roscoe	Annual herb	Muengueya samba (Kimb.)	Native	Shrubland savanna	4.5
37.	Caricaceae	*Carica papaya* L.	Tree/herb	Mamoeiro (Port.)	Cultivated	Cropland	4.5
38.	Asteraceae	*Tithonia diversifolia* (Hemsl.) A.Gray	Shrub	Cura-tudo (port.)	Introduced	Disturbed places	4.5
39.	Arecaceae	*Elaeis guineensis* (Jacq.)	Tree palm	Palmeira (Port.)	Naturalized	Cropland	4.5
40.	Apocynaceae	*Picralima nítida* (Stapf) T.Durand & H.Durand	Tree	Kikongo (Kimb.)	Introduced	Forest gallery	4.5
41.	Xanthorrhoeaceae	*Aloe ferox* Mill.	Herb succulent	Xandala (Kimb.)	Introduced	Cropland	4.5
42.	Amaryllidaceae	*Allium sativum* L.	Herb	Alho (Port.)	Cultivated	Cropland	4.5
43.	Poaceae	*Saccharum officinarum* L.	Grass	Cana de açúcar (Port.)	Cultivated	Cropland	4.5
44.	Amaranthaceae	*Spinacia oleracea* L.	Herb	Espinafre (Port)	Cultivated	Cropland	4.5
45.	Asteraceae	*Artemisia absinthium* L.	Shrub	Losna (Port.)	Native	Shrubland savanna	4.5
46.	Euphorbiaceae	*Manihot esculenta* Crantz	Shrub	Mandioqueira (Port.)	Cultivated	Cropland	4.5
47.	Euphorbiaceae	*Alchornea cordifolia* (Schumach. & Thonn.) Müll.Arg	Shrub	Mbunze (Kimb.)	Native	Forest gallery	4.5
48.	Solanaceae	*Capsicum annuum* L.	Herb	Gindungo (Port.)	Cultivated	Cropland	2.2
49.	Poaceae	*Cymbopogon citratus* (DC.) Stapf	Grass	Caxinde (Kimb.)	Cultivated	Cropland	2.2
50.	Myrtaceae	*Eugenia uniflora* L.	Shrub	Pitangueira (Port.)	Introduced	Cropland	2.2
51.	Malvaceae	*Abelmoschus esculentus* (L.) Moench.	Shrub	Quiabo (Port.)	Cultivated	Cropland	2.2
52.	Clusiaceae	*Garcinia mannii* Oliv.	Shrub	Ngadiadia (Kimb.)	Native	Forest gallery	2.2
53.	Asteraceae	*Acanthospermum hispidum* DC.	Herb	Kauenha (Kimb.)	Introduced	Disturbed places	2.2
54.	Anacardiaceae	*Mangifera indica* L.	Tree	Mangueira (Port.)	Cultivated	Cropland	2.2
55.	Moraceae	*Ficus thonningii* Blume	Tree	Lemba-lemba (Kimb.)	Native	Forest gallery	2.2
56.	Asteraceae	*Vernonia* sp	Herb	Kidiassute (Kimb.)	Introduced	Disturbed places	2.2
57.	Zingiberaceae	*Alpinia zerumbet* (Pers.) B.L. Burtt & R.M.Sm.	Herb	Colónia (Port.)	Introduced	ornamental	Nc
58.	Malvaceae	*Cola acuminata* (P. Beauv.) Schott & Endl.	Tree	Dikazo (Kimb.)	Native	Forest gallery	Nc
59.	Rubiaceae	*Coffea canephora* Pierre ex A.Froehner	Shrub	Cafeeiro (Port.)	Cultivated	Cropland	Nc
60.	Poaceae	*Zea mays* L.	Grass	Milheiro (Port.)	Cultivated	Cropland	Nc
61.	Nyctaginaceae	*Boerhavia diffusa* L.	Herb	Mutumbata (Kimb.)	Naturalized	Disturbed places	Nc
62.	Musaceae	*Musa paradisiaca* L.	Herb	Bananeira (Port.)	Cultivated	Cropland	Nc
63.	Lauraceae	*Cinnamomum verum* J.Presl.	Shrub	Canela (Port.)	Introduced	Cropland	Nc
64.	Lamiaceae	*Ocimum gratissimum* L.	Herb	Alfa vaca (Port.)	Introduced	Cropland	Nc
65.	Lamiaceae	*Mentha arvensis* L.	Herb	Hortelã (Port.)	Introduced	Cropland	Nc
66.	Cannabaceae	*Cannabis sativa* L.	Herb	Liamba (Port.)	Introduced	Cropland	Nc
67.	Asteraceae	*Ageratum conyzoides* L.	Herb	Cabuabuata (Kimb.)	Native	Disturbed places	Nc
68.	Apiaceae	*Foeniculum vulgare* Mill.	Herb	Funcho (Port.)	Cultivated	Cropland	Nc
69.	Anacardiaceae	*Spondias mombin* L.	Tree	Gajajeira (Port.)	Introduced	Cropland	Nc
70.	Viburnaceae	*Sambucus nigra* L.	Shrub	Sabugueiro (Port.)	Introduced	Cropland	Nc
71.	Fabaceae	*Pericopsis angolensis* (Baker) Meeuwen	Tree	Ngambo (Kimb.)	Native	Miombo/savanna	Nc
72.	Arecaceae	*Cocos nucifera* L.	Tree palm	Coqueiro (Port)	Introduced	Cropland	Nc
73.	Rosaceae	*Fragaria* sp.	Herb	Morangueiro (Port.)	Introduced	Cropland	Nc
74.	Lamiaceae	*Rosmarinus officinalis* L.	Herb	Alecrim (Port.)	Introduced	Cropland	Nc
75.	Fabaceae	*Arachis hypogaea* L.	Herb	Ginguba (Port.)	Cultivated	Cropland	Nc
76.	Malvaceae	*Sterculia dawei* Sprague	Tree	Mulende (Kimb.)	Native	Forest gallery	Nc
77.	Fabaceae	*Senna spectabilis* (DC.) H.S.Irwin & Barneby	Tree	Acacia (port.)	Introduced	Disturbed places	Nc
78.	Picrodendraceae	*Oldfieldia dactylophylla* (Welw. ex Oliv.) J.Léonard	Tree	Mubatangombe (Kimb.)	Native	Shrubland savanna/miombo	Nc

Abbreviations: Port, Portuguese; Kimb, Kimbundu.

Accurate recording of forest biodiversity offers a clearer picture of the conservation status of the most significant and frequently utilized species, thereby preventing agroforestry methods that could jeopardize ecosystem stability. In light of this, research (Fançony, 2021) has pointed out the disappearance of medicinal plants from their natural habitats in parts of Cuanza Sul, adjacent to Malanje. This loss stems from improper farming techniques, unregulated resource exploitation, and frequent fires, emphasizing the crucial need to safeguard these environments.

The study identified 78 species across 39 families (see Table 2), averaging approximately 2 species per family, which underscores a rich tapestry of botanical diversity in medicinal plants. Interestingly, twenty-one of these families (53.8%) are each represented by just one species. Additionally, three of the medicinal plants (1.1%) could only be classified down to the genus level. The family Fabaceae leads the list as the most frequently cited at 9%, followed by Asteraceae at 6.4%, and then Euphorbiaceae, Poaceae, and Zingiberaceae, each contributing 5.1%.

**TABLE 2:** Species distribution categorized based on family.

No.	Family	Number of species	Percent (%)
1.	Fabaceae	7	9
2.	Asteraceae	5	6.4
3.	Euphorbiaceae	4	5.1
4.	Poaceae	4	5.1
5.	Zingiberaceae	4	5.1
6.	Amaranthaceae	3	3.8
7.	Anacardiaceae	3	3.8
8.	Lamiaceae	3	3.8
9.	Malvaceae	3	3.8
10.	Myrtaceae	3	3.8
11.	Rubiaceae	3	3.8
12.	Solanaceae	3	3.8
13.	Amaryllidaceae	2	2.6
14.	Annonaceae	2	2.6
15.	Apocynaceae	2	2.6
16.	Arecaceae	2	2.6
17.	Lauraceae	2	2.6
18.	Phyllanthaceae	2	2.6
19.	Viburnaceae	1	1.3
20.	Apiaceae	1	1.3
21.	Asparagaceae	1	1.3
22.	Burseraceae	1	1.3
23.	Cannabaceae	1	1.3
24.	Caricaceae	1	1.3
25.	Celastraceae	1	1.3
26.	Clusiaceae	1	1.3
27.	Cochlospermaceae	1	1.3
28.	Combretaceae	1	1.3
29.	Costaceae	1	1.3
30.	Meliaceae	1	1.3
31.	Moraceae	1	1.3
32.	Moringaceae	1	1.3
33.	Musaceae	1	1.3
34.	Nyctaginaceae	1	1.3
35.	Picrodendraceae	1	1.3
36.	Polygalaceae	1	1.3
37.	Rosaceae	1	1.3
38.	Rutaceae	1	1.3
39.	Xanthorrhoeaceae	1	1.3

Ethnobotanical studies in Angola and elsewhere (Fançony, 2021; Felix et al., 2019; Gonçalves et al., 2019; Heinze et al., 2017; Malik et al., 2018; Novotna et al., 2020) have identified Fabaceae as a commonly used plant family. Plants with the highest ROP include *Terminalia brachystemma* (81.8), *Securidaca longepedunculata* (54.4), *Mondia whitei* (52.2), *Cochlospermum angolense* (34), *Mussaenda arcuata* (31.7), *Senna occidentalis* (29.4), *Aframomum alboviolaceum* (27.2), and *Monodora myristica* (27.1) (Table 1). Additionally, 22 plants were noted by only one informant each and lacked ROP calculations.

Drawing on the ethnopharmacological insights of our informants (see Table 3), we found that 17 plants are used to address a single ailment. Numerous plants integral to traditional medicine in Malanje exhibit diverse therapeutic applications. Particularly noteworthy are the seven plants with the highest ROP values, which have multiple medicinal purposes, including treatments for gastrointestinal issues and parasitic infections. Similar patterns were documented in Uíge (Lautenschläger et al., 2018; Heinze et al., 2017), where plants with highly specific applications are firmly embedded in cultural practice and valued for their perceived efficacy. Among the 78 botanically identified plants, an intriguing revelation is that 19 lack recorded medicinal uses in the existing literature, as highlighted in Table 3.

**TABLE 3:** Traditional knowledge of medicinal plants in Malanje.

No.	Scientific name	Traditional use	Plant parts used	Method of preparation	Application	Recorded literature uses	Reference
1.	*Terminalia brachystemma* Welw. ex Hiern	Open cervix, paludism, and stomachache	Root and leaf	Maceration	Oral, rectal, and vaginal	Antifungal (*in vitro*)	Liu et al. (2009), Masoko et al. (2005)
2.	*Securidaca longepedunculata* Fresen.	Inflammation, constipation, and stomachache	Root	Maceration	Topical	Anticonvulsants (*in vivo*)	Adeyemi et al. (2010)
3.	*Mondia whitei* (Hook.f.) Skeels	Stomachache, colic, and sexual impotence	Root	Maceration	Oral and inhalation	Antimalarials (*in vivo*)	Olanlokun et al. (2021)
4.	*Cochlospermum angolense* Welw. ex Oliv.	Hepatitis and paludism	Root	Maceration	Oral and topical	Anti-oxidant (*in vitro*)	Samba et al. (2025)
5.	*Mussaenda arcuata* Poir.	Bone pain, thrombosis, and labor pain	Leaf	Maceration	Topical	Not reported	​
6.	*Senna occidentalis* (L.) Link	Stomachache, paludism, snake bite, and hernia	Root and leaf	Decoction	Oral	Antidiabetic (*in vitro*)	Mishra et al. (2022) Nde et al. (2022)
7.	*Monodora myristica* (Gaertn.) Dunal	Stomachache, kyphosis, open cervix, and inflammation	Seed	Decoction	Oral and topical	Antidepressants (*in vivo*)	Ekeanyanwu et al. (2021)
8.	*Aframomum alboviolaceum* (Ridl.) K.Schum.	Abortion prevention and kyphosis	Root	Decoction	Oral	Not reported	​
9.	*Lannea welwitschii* (Hiern) Engl.	Fractures, toothache, and thrombosis	Stem	Cataplasm	Oral, topical, and rectal	Anti-proliferative (*in vitro*)	Saraux et al. (2023)
10.	*Xylopia aethiopica* (Dunal) A. Rich.	Stomachache, kyphosis, and open cervix	Seed	Decoction	Oral	Anti-cancer (*in vitro*)	Kuete et al. (2015)
11.	*Vachellia sieberiana* (DC.) Kyal. & Boatwr.	Inflammation, infertility, and backpain	Root	Maceration	Oral and topical	Not reported	​
12.	*Canarium schweinfurthii* Engl.	Stomachache, open cervix, typhoid fever, and hernia	Stem	Decoction	Oral	Termiticide (*in vivo*)	Nagawa et al. (2015)
13.	*Moringa oleífera* Lam.	Stomachache	Root, leaf, and seed	Decoction	Oral	Anti-inflammatory *(in vivo*)	Sulaiman et al. (2008)
14.	*Ricinus communis* L.	Headache, constipation, and thrombosis	Leaf and fruit	Decoction	Oral and topical	Phytoremediation (*in vitro*)	Yao et al. (2023)
15.	*Crossopteryx febrifuga* (Afzel. Ex G.Don) Benth.	Toothache and anemia	Leaf and root	Decoction	Oral and inhalation	Alzheimer (*in vitro*)	Tamfu et al. (2022) Maiga et al. (2006)
16.	*Cymbopogon densiflorus* Stapf	Kyphosis, hernia, and stomachache	Flower	Decoction	Oral	Asthma (*in vitro*)	Takaisi-Kikuni et al. (2000)
17.	*Eucalyptus globulus* Labill.	Asthma, tuberculosis, paludism, and cough	Leaf	Decoction	Inhalation, oral, and topical	Repellent (*in vitro)*	Pan et al. (2020)
18.	*Bauhinia forficata* Link	Sexual impotence and hepatitis	Root	Decoction	Oral	Anti-diabetes (*in vivo*)	Tonelli et al. (2022)
19.	*Dysphania ambrosioides* (L.) Mosyakin & Clemants	Stomachache, fever, epilepsy, hernia, and infertility	Leaf	Decoction	Oral, rectal, vaginal, and inhalational	Anti-inflammatory (*in vivo*)	Annaz et al. (2023)
20.	*Eriosema psoraleoides* (Lam.) G. Don	Sexual impotence, menstrual disorders, and intestinal infections	Root	Maceration	Oral and topical	Not reported	​
21.	*Gymnosporia senegalensis* Loes.	Cancer	Root	Maceration	Oral	Anti-inflammatory (*in vitro)*	Makgatho et al. (2018)
22.	*Asparagus sp.*	Stomachache and infertility	Root and leaf	Maceration	Oral	Cancer (*in vitro)*	Akissi et al. (2023)
23.	*Beta vulgaris* L.	Anemia	Root	Decoction	Oral	Testicular oxidative damage (*in vitro)*	Ojo et al. (2023)
24.	*Euphorbia pulcherrima* Willd. ex Klotzsch	Typhoid fever	Root	Decoction	Oral	Cancer (*in vitro*)	Moustak et al. (2018)
25.	*Aframomum melegueta* K. Schum.	Hemorrhoids	Seed	Maceration	Oral and topical	Not reported	​
26.	*Zingiber officinale* Rosc.	Sexual impotence, paludism, yellow fever, and thrombosis	Root	Decoction	Oral	Anti-inflammatory (*in vitro*)	Murugesan et al. (2020)
27.	*Allium cepa* L.	Inflammation, diabetes, injuries, flu, and hernia	Root	Infusion	Oral	Anti-inflammatory (*in vitro*)	Lee et al. (2023)
28.	*Nicotiana tabacum* L.	Hemorrhoids, asthma, and angina	Leaf	Decoction	Oral, topical, and sublingual	Anti-Alzheimer (*in vivo*)	Rawat et al. (2013)
29.	*Solanum lycopersicum* L.	Hemorrhage and headache	Leaf	Infusion	Oral and nasal	Anti-cancerogenic (*in* *vitro*)	Gerszberg et al. (2015)
30.	*Citrus limon* (L.) Burm. F.	Typhoid fever and paludism	Fruit	Decoction	Oral and topical	Anti-tumor (*in vitro*)	Zhou et al. (2021)
31.	*Phyllanthus niruri* L.	Kidney failure and kidney stones	Leaf	Decoction	Oral	Anti-inflammatory (*in vivo*)	Couto et al. (2013)
32.	*Psidium guajava* L.	Diarrhea and stomachache	Leaf	Decoction	Oral and inhalation	Hypertension (*in vivo*)	de Assis Braga et al. (2022)
33.	*Azadirachta indica* A. Juss.	Paludism and typhoid fever	Leaf	Decoction	Oral, inhalation, rectal, and topical	Hypertension (*in vitro)*	Iwuanyanwu et al. (2023)
34.	*Persea americana* Mill.	Hypertension, diarrhea, headache, and hernia	Leaf, fruit, and seed	Decoction	Oral, topical, and inhalation	Anti-tumor (*in vitro*)	Athaydes et al. (2022)
35.	*Hymenocardia acida* Tul.	Diarrhea, hemorrhoids, and psychosis	Root	Decoction	Oral	Hypertension (*in vivo*)	Manga et al. (2013)
36.	*Costus spiralis* (Jacq.) Roscoe	Urinary infection and sexual impotence	Stem	Decoction	Oral, vaginal, and topical (cataplasm)	Hypertension (*in vivo*)	de Farias Silva et al. (2021)
37.	*Carica papaya* L.	Constipation, headache, and facilitates childbirth	Root, leaf, and fruit	Decoction	Oral and inhalation	Dengue fever (*in vivo*)	Subenthiran et al. (2013)
38.	*Entada abyssinica* Steud.	Paludism, spinal pain, and sexual impotence	Leaf	Decoction	Oral	Not reported	​
39.	*Elaeis guineensis* Jacq.	Headache, burns, antidote, and prevents abortion	Fruit, flower, and stem	Maceration	Oral and inhalation	Healing activity (*in vivo*)	Rajoo et al. (2021)
40.	*Picralima nítida* (Stapf) T. Durand & H. Durand	Inflammation and bone pain	Stem	Maceration	Oral and topical (bath)	Not reported	​
41.	*Aloe ferox* Mill.	Typhoid fever, stomachache, and alopecia	Leaf	Maceration	Oral and topical	Antidiabetic (*in vitro*)	Mhaladi et al. (2016)
42.	*Allium sativum* L.	Inflammation, stomachache, and cough	Root	Maceration	Oral	Colon cancer (*in vitro*)	Liu et al. (2021)
43.	*Saccharum officinarum* L.	Heart failure and anemia	Leaf	Decoction	Oral	Hypertension (*in vitro*)	Cheng et al. (2021)
44.	*Spinacia oleracea* L.	Anemia	Leaf	Decoction	Oral	Not reported	​
45.	*Artemisia absinthium* L.	Respiratory diseases	Leaf	Decoction	Oral and topical (bath)	Focal cerebral ischemia (*in vitro)*	Bora and Sharma (2010)
46.	*Manihot esculenta* Crantz	Measles	Leaf	Maceration	Oral	Not reported	​
47.	*Alchornea cordifolia* (Schumach. & Thonn.) Müll.Arg	Fever	Root	Decoction	Topical, oral, and sublingual	Not reported	​
48.	*Capsicum annuum* L.	Epilepsy and labor pain	Root and leaf	Maceration	Oral	Anti-fungal (*in vitro*)	Arora et al. (2023)
49.	*Cymbopogon citratus* (DC.) Stapf	Cough, bladder pain, kidney obstruction, stomachache, and prevents abortion	Leaf	Decoction	Oral	Gastroprotective activity (*in vivo*)	Manvitha and Bidya (2014)
50.	*Eugenia uniflora* L.	Bloody diarrhea	Leaf	Decoction	Oral	Diuretic (*in vitro*)	Amorim et al. (2009)
51.	*Abelmoschus esculentus* (L.) Moench.	Glaucoma, conjunctivitis, and sexual impotence	Leaf and fruit	Infusion	Oral	Type 2 diabetes (*in vitro*)	Elkhalifa et al. (2021)
52.	*Garcinia mannii* Oliv.	Paludism and colic	Seed	Decoction	Oral	Not reported	​
53.	*Acanthospermum hispidum* DC.	Vaginal bleeding, paludism, diabetes, hepatitis, and antidote	Leaf	Decoction	Oral and topical	Antiviral (*in vitr*o)	Cantero-González et al. (2023)
54.	*Mangifera indica* L.	Abscess, hemorrhoids, and paludism	Leaf and Stem	Decoction	Oral and topical	Anti-inflammatory (*in vivo)*	Saviano et al. (2022)
55.	*Ficus thonningii* Blume	Hypertension	Leaf	Maceration	Oral	Diarrhea (*in vitro)*	Dangarembizi et al. (2013)
56.	*Vernonia sp*	Stomachache and hernia	Root	Decoction	Oral and inhalation	Diabetes (*in vitro*)	Dogra et al. (2020)
57.	*Alpinia zerumbet* (Pers.) B.L. Burtt & R.M.Sm.	Stomachache and bladder pain	Root	Infusion	Oral	Not reported	​
58.	*Cola acuminata* (P.Beauv.) Schott & Endl.	Nausea and vomiting	Fruit	Maceration	Oral	Alzheimer’s (*in vivo*)	Ishola et al. (2018)
59.	*Coffea canephora* Pierre ex A.Froehner	Diarrhea, wounds, and burns	Fruit	Infusion	Oral and topical	Not reported	​
60.	*Zea mays* L.	Abortion prevention	Stigmas	Decoction	Oral and topical	Not reported	​
61.	*Boerhavia diffusa* L.	Labor pain	Leaf	Maceration	Oral	Anti-inflammatory (*in vitro*)	Mathias et al. (2020)
62.	*Musa paradisiaca* L.	Prevention of abortion, hemorrhoids, and stimulation of labor	Leaf	Maceration	Oral	Anti-tumor (*in vivo*)	Lavudi et al. (2022)
63.	*Cinnamomum verum* J.Presl.	Stomachache and cough	Leaf	Decoction	Oral and topical	Anti-diabetic (*in vivo*)	Kwon et al. (2006)
64.	*Ocimum gratissimum* L.	Internal fever, hiccups, and cough	Leaf	Decoction	Oral, inhalation, topical, and sublingual	Anti-inflammatory (*in vivo*)	Shittu et al. (2016)
65.	*Mentha arvensis* L.	Stomachache and epilepsy	Root and leaf	Decoction	Oral	Analgesic (*in vivo*)	Biswas et al. (2014)
66.	*Cannabis sativa* L.	Psychosis, asthma, cough, bronchitis, nausea, vomiting, measles, and tuberculosis	Leaf	Decoction	Oral, rectal, and sublingual	Anti-fungal (*in vitro*)	Berardo et al. (2024)
67.	*Ageratum conyzoides* L.	Infertility	Leaf	Maceration	Oral, inhalation, and topical	Sleeping sickness (*in vitro*)	Nour et al. (2010)
68.	*Foeniculum vulgare* Mill.	Intestinal gas, flu, colic, kidney failure, and whooping cough	Root, stem, leaf, and flower	Decoction	Oral and topical	Gastroprotective (*in* *vitro*)	Jadid et al. (2023)
69.	*Spondias mombin* L.	Stomachache and hypotension	Root and fruit	Maceration	Oral and topical	Anti-inflammatory	Cabral et al. (2016)
70.	*Sambucus nigra* L.	Measles and epilepsy	Leaf and flower	Decoction	Oral and topical	Anti-inflammatory (*in vitro*)	Skowrońska et al. (2022)
71.	*Pericopsis angolensis* (Baker) Meeuwen	Thrombosis and bone fracture	Leaf and Root	Decoction	Oral	Not reported	​
72.	*Cocos nucifera* L.	Typhoid fever	Root	Decoction	Oral	Antifungal (*in vitro*)	Hernández-Flores et al. (2023)
73.	*Fragaria sp.*	Epilepsy	Root	Maceration	Oral and topical	Not reported	​
74.	*Rosmarinus officinalis* L.	Obesity	Leaf	Decoction	Oral, inhalation, and topical	Antifungal (*in vitro*)	Ghavam (2022)
75.	*Arachis hypogaea* L.	Headache	Seed	Cataplasm	Topical	Not reported	​
76.	*Sterculia dawei* Sprague	Kyphosis	Root	Maceration	Oral	Not reported	​
77.	*Senna spectabilis* (DC.) H.S. Irwin & Barneby	Diabetes, paludism, colic, diarrhea, and typhoid fever	Root and leaf	Decoction	Oral, inhalation, and topical	Trypanocidal activity (*in vitro*)	Lim et al. (2018)
78.	*Oldfieldia dactylophylla* (Welw. ex Oliv.) J.Léonard	Typhoid fever and open cervix	Root, stem, and leaf	Decoction	Oral	Not reported	​

Local informants consider most plants harmless, although a few come with noted side effects. For example, *Coffea canephora* might keep you up at night, *Cocos nucifera* can lead to constipation, *Nicotiana tabacum* could cause nausea and vomiting, and *Ricinus communis* is known for inducing diarrhea. Conversely, traditional healers point out that certain species, such as *Senna occidentalis*, *Terminalia brachystemma*, *Citrus limon*, *Phyllanthus niruri*, *Allium sativum*, *Solanum lycopersicum*, *Nicotiana tabacum*, *Securidaca longepedunculata*, *and Azadirachta indica*, have contraindications. Unfortunately, many people think that medicinal plants and natural remedies are harmless simply because they are “natural,” but this assumption is not accurate (Haq, 2004). Traditional practitioners mainly rely on culturally inferred undesirable effects, with adverse reactions often linked to sensory perceptions, particularly among newborns and pregnant women, who react to the strong odor and bitter taste as signs of toxicity. Ethnobotanical research among Brazilian communities has shown that local residents often have limited knowledge of the harmful potential of various plant species (Oler et al., 2019).

However, for this study, the recognition of contraindications by healers in Malanje suggests a meaningful level of empirical knowledge within local therapeutic practices. Their awareness of potential risks indicates a nuanced understanding of medicinal species, which is consistent with observations reported in other African ethnopharmacological studies. For example, *Senna occidentalis* has been associated with hepatotoxic effects when used over long periods (Adebayo et al., 2016), and *Securidaca longepedunculata* may become toxic when taken in excessive doses (Iwu 1997). These correspondences do not imply complete alignment but rather point to areas where local experience and scientific evidence converge, highlighting the potential value of integrating traditional knowledge into broader health and conservation discussions.

It is important to emphasize that, beyond the secondary metabolites that may render a species toxic, another crucial factor in assessing the safety of plant products is the contamination of forested regions, predominantly from heavy metals that medicinal plants can absorb (Asiminicesei et al., 2020; Sarma et al., 2012). Recent research has identified levels of heavy metals and organic pesticides in natural products exceeding regulatory limits (Karahan et al., 2020; Kohzadi et al., 2019; Kumar et al., 2018; Luo et al., 2021; Wang et al., 2019), underscoring the need for stringent quality controls on medicinal plant raw materials. Furthermore, mycotoxin contamination can compromise the quality and safety of these plant-based medicines, mainly when storage and preservation practices are inadequate (Ałtyn and Twarużek, 2020).

Therefore, assessing the efficacy and safety of these plants is crucial for their use in humans (Saad et al., 2017). The World Health Organization has emphasized the necessity of quality control for herbal medicines, especially regarding their safety (WHO, 2011; WHO, 2013). Recent ethnobotanical research highlights the toxic potential of traditional medicinal plants in Morocco (Kharchoufa et al., 2021).

In this vein, the Polytechnic Institute at Rainha Njinga a Mbande University is conducting pharmacognosic and phytochemical studies on the most commonly used traditional medicine plants in Malanje to address these concerns.

### Plant parts used in traditional medicine

**FIGURE 2:**
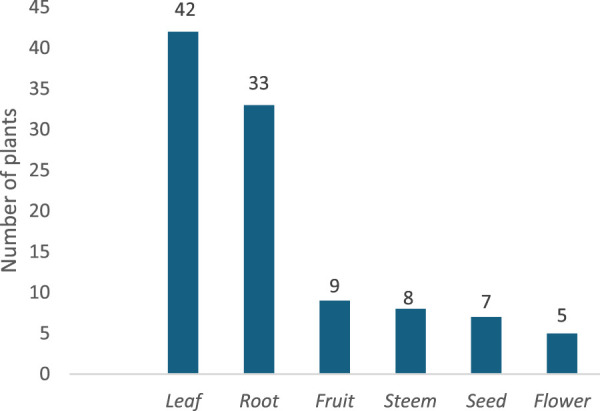
Plant components* traditionally utilized in Malanje. *In some plants, various parts are used for medicinal purposes.

Figure 2 reveals that leaves and roots are the most commonly utilized plant parts. Of the 78 plants identified, leaves take the lead, being used in 42 instances (53.8%). Roots, on the other hand, were mentioned in 33 cases (42.3%).

This preference likely stems not only from the medicinal properties of these plant parts but also from their year-round availability and ease of collection, unlike flowers or fruits, which are seasonal. Ethnobotanical studies frequently highlight this trend, showcasing leaves as a primary raw material (Cock and van Vuuren, 2020; Fançony, 2021; Gonçalves et al., 2019; Heinze et al., 2017; Malik et al., 2018; Varela et al., 2022). Moreover, research indicates the prominent use of roots in traditional medicine practices in regions such as Bibala, Namibe Province (Urso et al., 2016), and Kuito and Cuemba, Bié Province (Novotna et al., 2020).

The preference for using underground parts stems, in part, from the age-old belief passed down among medicinal practitioners that roots hold the richest concentration of healing substances and the most potent therapeutic qualities. However, overharvesting parts of medicinal plants that are crucial to their survival, such as roots, stems, and bark, constitutes destructive ethnopharmacological practice and significantly harms the floral reserves in regions plagued by overexploitation or poor management (Gonçalves et al., 2019). Although the conservation status of the recorded species indicated no immediate threats, comprehensive Red List assessments of Angolan flora remain limited. Therefore, it is crucial to raise awareness and provide training for traditional healers on sustainable harvesting practices, including substituting roots with less destructive plant parts, to preserve plant populations and sustain the continuity of traditional medicinal knowledge. Interestingly, many of these species offer alternative parts such as leaves and flowers, which might possess equally potent medicinal properties (Jena et al., 2017). In this vein, the historical documentation of Antunes and Dekindt, as reported by Tchamba et al. (2024), highlights their role in preserving traditional knowledge of medicinal plants in Angola and reveals a reduced dependence on roots among the population.

**FIGURE 3:**
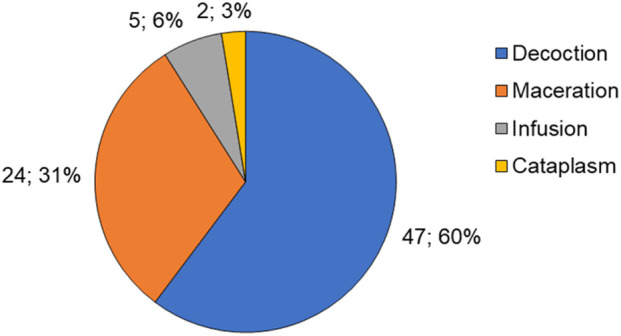
Extraction techniques utilized in traditional Malanje medicine.

The current research (Figure 3) revealed that decoction (60%) and maceration (31%) are the primary techniques for processing and extraction, similar to other ethnobotanical studies conducted in Angola (Novotna et al., 2020). These age-old methods predominantly use water as the solvent, with temperature being the key differentiator between them. Given this, extracts mostly capture high-polarity metabolites, which, unfortunately, restricts the therapeutic potential of the obtained extracts.

### Classification of treated pathologies (ICD-11)

The traditional medicine of Malanje often addresses three major conditions (Table 4): gastrointestinal issues at 43.6%, infectious or parasitic diseases at 29.5%, and a mix of symptoms—such as inflammation and fever—either tied to other illnesses or standing alone, making up 20.5%. Furthermore, 14.1% of the drug applications are geared toward treating conditions related to the genitourinary system, pregnancy, childbirth, and postnatal care. Interestingly, the medicinal plants identified in this research do not cater to disorders linked to the immune system, sleep problems, ear or mastoid issues, or neonatal conditions.

**TABLE 4:** Distribution of the diseases addressed by the medicinal plants mentioned, categorized according to ROP classes.

Treated pathology^a^	# Of plants based on ROP classes				Total	%
	Nc	0–24	25–49	50–100		
Infectious or parasitic diseases	5	15	2	1	23	29.5
Neoplasms	-	1	-	-	1	1.3
Diseases of the blood or blood-forming organs	-	4	-	-	4	5.1
Endocrine, nutritional, or metabolic diseases	3	2	-	-	5	6.4
Mental, behavioral, or neurodevelopmental disorders	1	1	-	-	2	2.6
Diseases of the nervous system	4	7	-	-	11	14.1
Diseases of the visual system	-	1	-	-	1	1.3
Diseases of the circulatory system	2	6	-	-	8	10.3
Diseases of the respiratory system	4	5	-	-	9	11.5
Diseases of the digestive system	8	21	2	3	34	43.6
Diseases of the skin	-	1	-	-	1	1.3
Diseases of the musculoskeletal system or connective tissue	2	3	2	-	7	9.0
Diseases of the genitourinary system	3	8	-	-	11	14.1
Conditions related to sexual health	-	6	-	1	7	9.0
Pregnancy, childbirth, or the puerperium	4	4	2	1	11	14.1
Injury, poisoning, or certain other consequences of external causes	-	3	1	-	4	5.1
Symptoms, signs, or clinical findings not elsewhere classified	3	10	2	1	16	20.5

Nc, ROP not calculated.

^a^ International Classification of Diseases. Adapted from “ICD-11: International Classification of Diseases (11th revision),” World Health Organization (WHO, 2019). Retrieved from https://icd.who.int/browse11/l-m/en.

These findings likely reflect the real-world prevalence of certain ailments, such as malaria and typhoid fever, which have high rates not just in Malanje but across Angola in general (WHO, 2018).

### Comparative analysis of ethnopharmacological and pharmacological evidence

The ethnopharmacological register compiled in Malanje brings to light plant species of notable cultural and therapeutic relevance. Among those most consistently prioritized by local healers are *Terminalia brachystemma*, *Securidaca longepedunculata*, *Mondia whitei*, *Cochlospermum angolense*, *Aframomum alboviolaceum*, *and Monodora myristica*. A comparative reading alongside pharmacological literature reveals compelling convergence while exposing evidence gaps that merit systematic investigation.

*Terminalia brachystemma* (Mueia), widely employed for gastrointestinal complaints, aligns with findings on related Terminalia species demonstrating antimicrobial, antioxidant, and hepatoprotective activity, as reported by Joujeh and Joujeh (2023). Nonetheless, Das et al. (2020) document cytotoxicity at high concentrations, pointing to the need for careful dose–response and safety evaluations. Similarly, *Securidaca longepedunculata* (Mutungo), highly valued for managing fever and pain, is supported by evidence of analgesic, anti-inflammatory, antimalarial, and antimicrobial effects summarized by Abubakar et al. (2019). Yet, concerns regarding neurotoxicity and high-dose toxicity in root–bark extracts, highlighted by Mongalo et al. (2015), remain insufficiently addressed in community practice.

In the case of *Mondia whitei* (Mundondo), healers describe its use as a tonic and digestive aid. Scientific studies expand this profile: Hagari and Nguta (2025) and Deeh et al. (2024) document aphrodisiac, antioxidant, antimicrobial, and anti-stress activities, indicating promising therapeutic breadth, although systematic toxicity assessments and clinical studies remain limited. *Cochlospermum angolense (*Mbrututo), traditionally used for hepatic disorders and malaria prophylaxis, has been supported by recent analyses reporting strong antioxidant and hepatoprotective effects, including works by Samba et al. (2025) and Hossi (2017). However, as with many medicinal species, rigorous toxicity evaluations and clinical-outcome research remain scarce.

*Aframomum alboviolaceum* (Ginguenga), prescribed for digestive and infectious conditions, is distinguished by its essential-oil richness. Literature reviews by Inkoto et al. (2021) and Ma et al. (2025) highlight anti-sickling, antimicrobial, antimalarial, and anti-inflammatory properties, although variability in traditional preparation methods continues to complicate alignment with pharmacological findings. Finally, *Monodora myristica* (Jipepe), employed for gastrointestinal and infectious disorders, exhibits antioxidant, antihypertensive, hepatoprotective, antibacterial, antifungal, and neuroprotective effects, as demonstrated by Agiriga and Siwela (2017) and Isiogugu et al. (2018). Although seed and bark extracts appear safe at typical traditional doses, reports suggest that high concentrations may influence lipid metabolism.

Taken together, these observations illustrate that traditional knowledge in Malanje frequently converges with pharmacological evidence, particularly for the management of gastrointestinal and infectious disease while highlighting persistent gaps concerning toxicity, dosage standardization, and clinically validated outcomes. The register, therefore, serves not only as culturally grounded documentation of local therapeutic practices but also as a strategic shortlist of priority species for future pharmacological, toxicological, and clinical research. Integrating ethnographic insights with biomedical inquiry can support preservation, rigorous evaluation, and potential incorporation of traditional remedies in ways that strengthen both community healthcare practice and national health strategies.

### Relevance in the context of Malanje municipality

Despite the ecological potential, the Malanje region has remained largely absent from ethnopharmacological surveys, leaving a critical gap in Angola’s national knowledge base. From a sociocultural perspective, Malanje is predominantly inhabited by the Kimbundu ethnolinguistic group, whose oral traditions serve as the primary vehicle for transmitting medicinal knowledge. Marketplaces such as Cangambo function as hubs for the circulation of remedies and therapeutic practices, underscoring the importance of documenting this knowledge before it is eroded by urbanization, generational shifts, and high levels of illiteracy among healers. Recording local names, practices, and preparation methods not only preserves cultural identity but also strengthens intergenerational transmission of knowledge.

### Healthcare relevance

The ethnopharmacological register compiled in Malanje Municipality offers significant potential to strengthen healthcare delivery at both community and institutional levels. By documenting 272 ethnospecies and identifying 78 botanical species across 39 families, the study provides a structured baseline of traditional practices that directly address the most prevalent local health concerns. The predominance of remedies for gastrointestinal disorders and infectious or parasitic diseases reflects the burden of disease in the region, underscoring the relevance of this knowledge to primary care. High-priority species such as *Terminalia brachystemma*, *Securidaca longepedunculata*, and *Mondia whitei* emerge as candidates for pharmacological validation, conservation, and possible integration into standardized phytomedicines. Moreover, the register supports Angola’s National Policy for Traditional and Complementary Medicine by providing evidence to guide the safe, culturally sensitive incorporation of traditional practices into formal health systems.

### Validity of the information

The validity of this information lies primarily in its ethnographic and cultural dimensions. Systematic interviews, participatory observation, and taxonomic verification ensure that the register accurately reflects community knowledge and therapeutic priorities. The use of ROP adds quantitative weight, highlighting species most valued by healers. However, the register’s validity as clinical evidence remains limited. ROP measures popularity and fidelity of use rather than proven efficacy, and the absence of standardized dosage, toxicity profiles, and clinical outcome data prevents direct translation into biomedical practice. Thus, while the register is reliable as a cultural and ethnobotanical baseline, it must be regarded as exploratory evidence requiring further pharmacological and clinical validation before therapeutic claims can be generalized.

## Limitations and future directions

Several limitations must be acknowledged. The sample size was relatively small (20 healers) and concentrated in specific markets and neighborhoods, potentially underrepresenting the diversity of practices across rural and peri-urban areas. Although 272 ethnospecies were recorded, only 78 were identified to the species level, leaving a substantial proportion of the local pharmacopoeia taxonomically unresolved. Furthermore, preparation methods varied among healers, and no standardized dosages or toxicity data were collected, constraining the ability to assess risks and benefits. Although this study provides an initial register of medicinal plants in Malanje, much remains to be done. Future research could broaden sampling to include healers from other municipalities across the province, thereby offering a more representative picture of local practices. Completing taxonomic identification and undertaking pharmacognosic and phytochemical analyses of high-ROP species, such as *Terminalia brachystemma*, *Securidaca longepedunculata*, and *Mondia whitei*, would help clarify their bioactive constituents and therapeutic potential. Careful safety assessments, including toxicity assays and contaminant screening, are also needed to better understand risk thresholds and ensure community wellbeing. Conservation strategies, including the possible cultivation of high-demand species, may help reduce pressure on wild populations. In addition, policy-oriented research could explore ways of aligning ethnopharmacological findings with Angola’s National Policy for Traditional and Complementary Medicine while ensuring that traditional knowledge holders are respected and benefit fairly from any future bioprospecting or product development.

As part of the medicinal plants initiative at the Polytechnic Institute of Rainha Njinga a Mbande University, steps are being taken toward the creation of a herbarium for specimen storage. Although this stage has not yet been reached, our team has worked alongside local healers to encourage sustainable plant collection practices and fire-prevention techniques. We have also established communication with local authorities to share information on the municipality’s biodiversity potential and discuss collective strategies to preserve its ecological diversity. GPS coordinates for each species have been carefully recorded, providing a resource for future monitoring and conservation efforts. These are modest beginnings, but they represent important steps toward safeguarding both biological and cultural heritage in Malanje.

## Data Availability

The raw data supporting the conclusions of this article will be made available by the authors, without undue reservation.
