# Supplementary material for: An ethnopharmacological assessment of medicinal plants in Malanje Municipality, Angola
Source: Front Pharmacol. 2026 Feb 25;16:1702353. doi: 10.3389/fphar.2025.1702353 (PMC12980091; doi:10.3389/fphar.2025.1702353)
Supplement: Supplementary file 1 [file DataSheet1.pdf]

No.	Family	Scientific name	habitus	Common name	Distribution data for each species	Collecting Place	ROP
79.	-	-	Herb	Kaluma Mungombe (Kimb.)	-	Cropland	40,50
80.	-	-	Tree	Betama (Kimb.)	-	Cropland	36,73
81.	-	-	Shrub	Canjugo (Kimb.)	-	Cropland	36,33
82.			Tree	Huiua (Kimb.)	-	Cropland	36,00
83.	-	-	Shrub	Cacoricori (Kimb.)	-	Cropland	34,72
84.	-	-	Tree	Calhandagi (Song.)	-	Cropland	33,33
85.	-	-	Shrub	Katokola (Kimb.)	-	Cropland	33,33
86.	-	-	Tree	Cassamba-mboa (Kimb.)	-	disturbed places	33,33
87.	-	-	Tree	Canhanga (Kimb.)	-	Cropland	32,65
88.	-	-	Tree	CadiegiCamox (Kimb.)	-	Cropland	32,00
89.	-	-	Tree	Cassuto (Kimb.)	-	Cropland	31,25
90.	-	-	Tree	Kafalacombe (Kimb.)	-	Cropland	29,75
91.	-	-	Tree	Calhamacuba (Kimb.)	-	Cropland	29,63
92.	-	-	Herb	Diez (Kimb.)	-	Cropland	29,17
93.	-	-	Shrub	Kananga (Kimb.)	-	Cropland	28,93
94.	-	-	Tree	Baze-mbaze (Kimb.)	-	Cropland	28,44
95.	-	-	Tree	Cajinga (Kimb.)	-	Cropland	27,55
96.	-	-	Herb	Fita (Kimb.)	-	disturbed places	26,67
97.	-	-	Herb	Dibubulo (Kimb.)	-	Cropland	26,59
98.	-	-	Shrub	Cabobó (Kimb.)	-	Cropland	26,45
99.	-	-	Shrub	Fulombunda (Kimb.)	-	Cropland	25,93
100.	-	-	Herb	Catape (Kimb.)	-	Cropland	25,40
101.	-	-	Tree	Caminomino (Kimb.)	-	Cropland	25,00
102.	-	-	Tree	Camohafo (Kimb.)	-	Cropland	25,00
103.	-	-	Tree	Dungue (Kimb.)	-	Cropland	25,00
104.	-	-	Tree	Gimbombo (Kimb.)	-	Cropland	25,00
105.	-	-	Tree	Jinzunzo (Kimb.)	-	Cropland	25,00

106.	-	-	Tree	Kafulakuembe (Kimb.)	-	Cropland	25,00
107.	-	-	Tree	Kahondi (Kimb.)	-	Cropland	25,00
108.	-	-	Herb	Katikassassa (Kimb.)	-	Cropland	25,00
109.	-	-	Herb	Ngongovirou (Kimb.)	-	Cropland	25,00
110.	-	-	Herb	Peta (Kimb.)	-	Cropland	25,00
111.	-	-	Herb	Calula (Kimb.)	-	Cropland	25,00
112.	-	-	Herb	Camoxindi Coxi (Kimb.)	-	Cropland	24,79
113.	-	-	Shrub	Capulamalonga (Kimb.)	-	disturbed places	24,69
114.	-	-	Tree	Gergelim (Kimb.)	-	disturbed places	24,69
115.	-	-	Shrub	Camucundo (Kimb.)	-	Cropland	24,49
116.	-	-	Herb	Canzela (Kimb.)	-	disturbed places	24,49
117.	-	-	Tree	Camba Camaso (Kimb.)	-	Cropland	24,00
118.	-	-	Tree	Guenga Lufeto (Kimb.)	-	disturbed places	24,00
119.	-	-	Shrub	Dilembo (Kimb.)	-	Cropland	23,44
120.	-	-	Herb	Catundu (Kimb.)	-	Cropland	23,44
121.	-	-	Herb	Caixa (Kimb.)	-	disturbed places	22,22
122.	-	-	Tree	Camuenha (Kimb.)	-	Cropland	22,22
123.	-	-	Tree	Canema (Kimb.)	-	Cropland	22,22
124.	-	-	Tree	Capulamalonga (Kimb.)	-	Cropland	22,22
125.	-	-	Tree	Kakiti (Kimb.)	-	disturbed places	22,22
126.	-	-	Tree	Digidia (Kimb.)	-	Cropland	22,22
127.	-	-	Tree	Kacangatuya (Kimb.)	-	Cropland	21,43
128.	-	-	Tree	Camucamba (Kimb.)	-	Cropland	21,30
129.	-	-	Herb	Cafilibunda (Kimb.)	-	Cropland	20,41
130.	-	-	Shrub	Caialacamoxi (Kimb.)	-	Cropland	20,83
131.	-	-	Herb	Cahuihua (Kimb.)	-	Cropland	20,41
132.	-	-	Tree	Kancundocahoxi (Kimb.)	-	Cropland	20,00

133.	-	-	Herb	Cafuba coroto (Kimb.)	-	Cropland	19,83
134.	-	-	Tree	Fuba Calote (Kimb.)	-	Cropland	19,05
135.	-	-	Shrub	Citumbi (Kimb.)	-	Cropland	18,75
136.	-	-	Tree	Hondembe (Kimb.)	-	Cropland	18,75
137.	-	-	Tree	Cacaxi (Kimb.)	-	disturbed places	18,75
138.	-	-	Tree	Kaditanga Coxi (Kimb.)	-	disturbed places	18,52
139.	-	-	Tree	Dicudila (Kimb.)	-	disturbed places	17,28
140.	-	-	Tree	Indai (Kimb.)	-	Cropland	16,67
141.	-	-	Herb	Kariandagi (Kimb.)	-	Cropland	16,67
142.	-	-	Shrub	Cafungo (Kimb.)	-	Cropland	16,67
143.	-	-	Shrub	Caluculo (Kimb.)	-	disturbed places	16,67
144.	-	-	Herb	Cambondo (Kimb.)	-	disturbed places	16,67
145.	-	-	Tree	Canzomzó (Kimb.)	-	Cropland	16,67
146.	-	-	Herb	Capetele (Kimb.)	-	disturbed places	16,67
147.	-	-	Shrub	Cassabão (Kimb.)	-	Cropland	16,67
148.	-	-	Tree	Kueza (Kimb.)	-	Cropland	16,50
149.	-	-	Shrub	Cabuquila (Kimb.)	-	Cropland	16,33
150.	-	-	Herb	Carrimba Cocota (Kimb.)	-	Cropland	16,33
151.	-	-	Herb	Dimuemueta (Kimb.)	-	Cropland	16,00
152.	-	-	Tree	Cacama (Kimb.)	-	Cropland	16,00
153.	-	-	Herb	Kacululo (Kimb.)	-	Cropland	16,00
154.	-	-	Tree	Kidiata (Kimb.)	-	Cropland	16,00
155.	-	-	Herb	Towele/ Touele (Kimb.)	-	Cropland	16,00
156.	-	-	Tree	Camuxidica lundo (Kimb.)	-	Cropland	16,00
157.	-	-	Herb	Berinjela (Port.)	-	disturbed places	15,32
158.	-	-	Herb	Zamba (Kimb.)	-	Cropland	15,00
159.	-	-	Tree	Cananguelund o (Kimb.)	-	Cropland	15,00

160.	-	-	Herb	Bióio (Kimb.)	-	Cropland	15,00
161.	-	-	Shrub	Colamangombe/ Calamagombe (Kimb.)	-	Cropland	15,00
162.	-	-	Tree	Cabaça (Kimb.)	-	Cropland	14,88
163.	-	-	Shrub	Camondu (Kimb.)	-	disturbed places	14,81
164.	-	-	Herb	Canombecatucula (Kimb.)	-	Cropland	14,81
165.	-	-	Herb	Dibata (Kimb.)	-	Cropland	14,74
166.	-	-	Herb	Sissinhossinho (Kimb.)	-	Cropland	14,22
167.	-	-	Shrub	Cadicundula (Kimb.)	-	Cropland	14,06
168.	-	-	Shrub	Kanhanga (Kimb.)	-	disturbed places	13,22
169.	-	-	Shrub	Cabeia (Kimb.)	-	Cropland	12,50
170.	-	-	Tree	Cacodicodi (Kimb.)	-	disturbed places	12,50
171.	-	-	Tree	Caminhominho (Kimb.)	-	Cropland	12,50
172.	-	-	Herb	Diambambe (Kimb.)	-	Cropland	12,50
173.	-	-	Tree	Camuxidi-catóco (Kimb.)	-	Cropland	12,50
174.	-	-	Herb	Candona (Kimb.)	-	disturbed places	12,50
175.	-	-	Tree	Catanquela (Kimb.)	-	Cropland	12,50
176.	-	-	Tree	Chava (Kimb.)	-	Cropland	12,50
177.	-	-	Tree	Gulo (Kimb.)	-	Cropland	12,50
178.	-	-	Shrub	Gipanda (Kimb.)	-	Cropland	12,50
179.	-	-	Herb	Cangululo (Kimb.)	-	Cropland	12,00
180.	-	-	Shrub	Dihamba (Kimb.)	-	Cropland	12,00
181.	-	-	Herb	Cassalanhala (Kimb.)	-	Cropland	12,00
182.	-	-	Herb	Kalunbonbo (Kimb.)	-	Cropland	12,00
183.	-	-	Tree	Ditondo/ Migosta (Kimb.)	-	Cropland	12,00
184.	-	-	Tree	Ganga Ilamba (Kimb.)	-	Cropland	12,00
185.	-	-	Shrub	Jungo (Kimb.)	-	Cropland	12,00

186.	-	-	Herb	Buma (Kimb.)	-	Cropland	11,11
187.	-	-	Tree	Cavucovuco (Kimb.)	-	Cropland	11,11
188.	-	-	Tree	Dele (Kimb.)	-	disturbed places	11,11
189.	-	-	Shrub	Chibutamo (Kimb.)	-	disturbed places	11,11
190.	-	-	Tree	Cabungatango (Kimb.)	-	disturbed places	11,11
191.	-	-	Tree	Capim (Kimb.)	-	Cropland	11,11
192.	-	-	Tree	Dixixite (Kimb.)	-	Cropland	11,11
193.	-	-	Tree	Giquesso (Kimb.)	-	Cropland	11,00
194.	-	-	Shrub	Diassonde (Kimb.)	-	Cropland	8,64
195.	-	-	Herb	Dihodi (Kimb.)	-	disturbed places	8,33
196.	-	-	Tree	Dilemba-sende (Kimb.)	-	Cropland	8,33
197.	-	-	Shrub	Hocemanagoji (Kimb.)	-	Cropland	8,33
198.	-	-	Tree	Dimi-diangombe (Kimb.)	-	disturbed places	8,33
199.	-	-	Shrub	Gikefo (Kimb.)	-	disturbed places	8,33
200.	-	-	Herb	Katindo (Kimb.)	-	disturbed places	8,33
201.	-	-	Shrub	Cambati/ camba (Kimb.)	-	Cropland	8,33
202.	-	-	Shrub	Catetele (Kimb.)	-	Cropland	8,33
203.	-	-	Tree	Dibunguede (Kimb.)	-	Cropland	8,26
204.	-	-	Shrub	Dixite (Kimb.)	-	Cropland	8,00
205.	-	-	Tree	Guife (Kimb.)	-	disturbed places	8,00
206.	-	-	Tree	Pondela (Kimb.)	-	Cropland	8,00
207.	-	-	Herb	Caifata (Kimb.)	-	Cropland	8,00
208.	-	-	Shrub	Cassacala (Kimb.)	-	Cropland	8,00
209.	-	-	Tree	Catchutchu (Kimb.)	-	Cropland	8,00
210.	-	-	Tree	Camuelele (Kimb.)	-	disturbed places	8,00
211.	-	-	Tree	Cinzenze (Kimb.)	-	Cropland	8,00
212.	-	-	Tree	Dihela (Kimb.)	-	Cropland	6,25
213.	-	-	Tree	Gamba (Kimb.)	-	disturbed places	6,25

214.	-	-	Tree	Inxilo (Kimb.)	-	Cropland	6,25
215.	-	-	Tree	Capandi (Kimb.)	-	Cropland	6,25
216.	-	-	Shrub	Ndangiluandelé (Kimb.)	-	Cropland	6,25
217.	-	-	Tree	Wemba-Wemba (Kimb.)	-	Cropland	6,25
218.	-	-	Shrub	Bilolo (Kimb.)	-	Cropland	6,25
219.	-	-	Herb	Cambuengue (Kimb.)	-	disturbed places	6,25
220.	-	-	Tree	DangeLundondo (Kimb.)	-	disturbed places	6,25
221.	-	-	Tree	Chitungo (Kimb.)	-	Cropland	6,25
222.	-	-	Tree	Kamunganhaen (Kimb.)	-	disturbed places	5,56
223.	-	-	Shrub	Corongaixa (Kimb.)	-	Cropland	5,56
224.	-	-	Herb	Kangambocatóco (Kimb.)	-	Cropland	5,56
225.	-	-	Tree	Cesse (Kimb.)	-	disturbed places	5,56
226.	-	-	Herb	Cipoepoe (Song.)	-	Cropland	5,56
227.	-	-	Tree	Kakumbikanganga (Kimb.)	-	Cropland	3,13
228.	-	-	Tree	Ndemba ia maji (Kimb.)	-	Cropland	1,25
229.	-	-	Tree	Sende-lua-ponde (Kimb.)	-	Cropland	0,82
230.	-	-	Shrub	Bula-tadi (Kimb.)	-	disturbed places	0,50
231.	-	-	Tree	Capeixe (Kimb.)	-	Cropland	0,50
232.	-	-	Tree	Catchiringuecatchimue (Kimb.)	-	Cropland	0,50
233.	-	-	Herb	Calissange (Song.)	-	disturbed places	0,33
234.	-	-	Shrub	Chinxi (Kimb.)	-	Cropland	0,33
235.	-	-	Shrub	Dizala (Kimb.)	-	Cropland	0,25
236.	-	-	Tree	Catore (Kimb.)	-	Cropland	0,25
237.	-	-	Tree	Holomuto (Kimb.)	-	disturbed places	0,21
238.	-	-	Tree	Dito dia Mbande	-	Cropland	0,20

				(Kimb.)			
239.	-	-	Shrub	Kambuengue (Kimb.)	-	Cropland	0,19
240.	-	-	Tree	Calualualua (Kimb.)	-	Cropland	0,17
241.	-	-	Herb	Capopotela (Kimb.)	-	disturbed places	0,15
242.	-	-	Herb	Chefe (Port.)	-	disturbed places	0,14
243.	-	-	Herb	Caluzo (Kimb.)	-	Cropland	0,13
244.	-	-	Shrub	Cateta (Kimb.)	-	disturbed places	0,13
245.	-	-	Tree	Divuri (Kimb.)	-	Cropland	0,11
246.	-	-	Tree	Diquelesse (Kimb.)	-	Cropland	0,08
247.	-	-	Tree	Caiongo-cutecute (Kimb.)	-	Cropland	0,06
248.	-	-	Herb	Camundo Caboye (Kimb.)	-	Cropland	0,03
249.	-	-	Herb	Dilolobiúlo (Kimb.)	-	Cropland	NC
250.	-	-	Shrub	Dituta (Kimb.)	-	Cropland	NC
251.	-	-	Shrub	Doce-Doce (Kimb.)	-	disturbed places	NC
252.	-	-	Herb	Futacui (Kimb.)	-	Cropland	NC
253.	-	-	Tree	Gibatamba (Kimb.)	-	Cropland	NC
254.	-	-	Tree	Gonongono (Kimb.)	-	disturbed places	NC
255.	-	-	Tree	Guiniamacung a (Kimb.)	-	Cropland	NC
256.	-	-	Shrub	Huxila (Kimb.)	-	Cropland	NC
257.	-	-	Shrub	Inkia (Kimb.)	-	Cropland	NC
258.	-	-	Tree	Jindão (Kimb.)	-	Cropland	NC
259.	-	-	Shrub	Jinzeu (Kimb.)	-	disturbed places	NC
260.	-	-	Herb	Kastigo (Kimb.)	-	Cropland	NC
261.	-	-	Tree	Mulhacala (Kimb.)	-	Cropland	NC
262.	-	-	Herb	Amb (Kimb.)	-	Cropland	NC
263.	-	-	Herb	Bulo (Kimb.)	-	Cropland	NC
264.	-	-	Shrub	Cacate (Kimb.)	-	Cropland	NC
265.	-	-	Herb	Cacolo (Kimb.)	-	Cropland	NC

266.	-	-	Herb	Cadimuinaimbanda (Kimb.)	-	Cropland	NC
267.	-	-	Tree	Cambeia (Kimb.)	-	Cropland	NC
268.	-	-	Shrub	Caxiricaxi (Kimb.)	-	Cropland	NC
269.	-	-	Herb	Chilongo (Song.)	-	Cropland	NC
270.	-	-	Herb	Cipoveve (Kimb.)	-	Cropland	NC
271.	-	-	Shrub	Diadiaxe (Kimb.)	-	Cropland	NC
272.	-	-	Tree	Diboque (Kimb.)	-	Cropland	NC
