# Supplementary material for: An ethnopharmacological assessment of medicinal plants in Malanje Municipality, Angola
Source: Front Pharmacol. 2026 Feb 25;16:1702353. doi: 10.3389/fphar.2025.1702353 (PMC12980091; doi:10.3389/fphar.2025.1702353)
Supplement: Supplementary file 2 [file DataSheet2.docx]

**Survey Form Nº**

Section A:

| Data | Location of activities | Gender | | Age | When you began (year) | Educational Level | | | |
| --- | --- | --- | --- | --- | --- | --- | --- | --- | --- |
|  |  | M | F |  |  |  |  |  |  |
|  |  |  |  |  |  | Iliterate | Primary | Secondary | Academic |
|  |  |  |  |  |  |  |  |  |  |

Section B1:

| Plant  Nº___ | Trivial name: | | | | Botanical classification  Plant name (family): | | |
| --- | --- | --- | --- | --- | --- | --- | --- |
|  | Utilization  (Type of desease) |  | | | | | |
|  | Extraction techniques | Decotion | Maceration | Infusion | | Cataplasm | |
|  |  |  |  |  | |  | |
|  | Plant component | Leaf | Root | Steem | Fruit | Flower | Stigmas |
|  |  |  |  |  |  |  |  |
|  | Collateral effects |  | | | | | |
|  | Contraindications |  | | | | | |

Section B2:

| Location Area |  |
| --- | --- |
| GPS |  |
